# Unsupervised semi-automated MRI segmentation detects cortical lesion expansion in chronic traumatic brain injury

**DOI:** 10.3389/fneur.2025.1640514

**Published:** 2025-10-15

**Authors:** Holly J. Freeman, Alexander S. Atalay, Jian Li, Evie Sobczak, Natalie Gilmore, Samuel B. Snider, Brian C. Healy, Holly Carrington, Enna Selmanovic, Ariel Pruyser, Lisa Bura, David P. Sheppard, David Hunt, Alan C. Seifert, Yelena G. Bodien, Jeanne M. Hoffman, Christine L. Mac Donald, Kristen Dams-O'Connor, Brian L. Edlow

**Affiliations:** ^1^Department of Neurology, Center for Neurotechnology and Neurorecovery, Massachusetts General Hospital and Harvard Medical School, Boston, MA, United States; ^2^Department of Radiology, Athinoula A. Martinos Center for Biomedical Imaging, Massachusetts General Hospital and Harvard Medical School, Boston, MA, United States; ^3^Department of Neurology, Brigham and Women's Hospital and Harvard Medical School, Boston, MA, United States; ^4^Massachusetts General Hospital Biostatistics Center, Massachusetts General Hospital, Boston, MA, United States; ^5^Ann Romney Center for Neurologic Diseases, Brigham and Women's Hospital, Boston, MA, United States; ^6^Department of Rehabilitation and Human Performance, Icahn School of Medicine at Mount Sinai, New York, NY, United States; ^7^Friedman Brain Institute, Icahn School of Medicine at Mount Sinai, New York, NY, United States; ^8^Department of Rehabilitation Medicine, University of Washington School of Medicine, Seattle, WA, United States; ^9^Department of Neurological Surgery, University of Washington, Seattle, WA, United States; ^10^Biomedical Engineering and Imaging Institute, Department of Radiology, Icahn School of Medicine at Mount Sinai, New York, NY, United States; ^11^Department of Physical Medicine and Rehabilitation, Spaulding Rehabilitation Hospital, and Harvard Medical School, Charlestown, MA, United States; ^12^Department of Neurology, Icahn School of Medicine at Mount Sinai, New York, NY, United States; ^13^Department of Neuroscience, Icahn School of Medicine at Mount Sinai, New York, NY, United States

**Keywords:** traumatic brain injury, cortical lesion, segmentation, longitudinal MRI, semi-automated

## Abstract

Traumatic brain injury (TBI) is a risk factor for neurodegeneration and cognitive decline, yet the underlying pathophysiologic mechanisms are incompletely understood. This gap in knowledge is in part related to a lack of reliable and efficient methods for measuring cortical lesions in neuroimaging studies. The objective of this study was to develop a semi-automated lesion detection tool and apply it to an investigation of longitudinal changes in brain structure among individuals with chronic TBI. We identified 24 individuals with chronic moderate-to-severe TBI enrolled in the Late Effects of TBI (LETBI) study who had cortical lesions detected by T1-weighted MRI and underwent two MRI scans at least 2 years apart. Initial MRI scans were performed more than 1 year post-injury, and follow-up scans were performed a median of 3.1 (IQR = 1.7) years later. We leveraged FreeSurfer parcellations of T1-weighted MRI volumes and a recently developed super-resolution technique, SynthSR, to automate the identification of cortical lesions in this longitudinal dataset. Trained raters received the data in a randomized order and manually edited the automated lesion segmentations, yielding a final semi-automated lesion mask for each scan at each time point. Inter-rater variability was assessed in an independent cohort of 10 additional LETBI subjects with cortical lesions. The semi-automated lesion segmentations showed a high level of accuracy compared to “ground truth” lesion segmentations performed via manual segmentation by a separate blinded rater. In a longitudinal analysis of the semi-automated segmentations, lesion volume increased between the two time points with a median volume change of 4.91 (IQR = 12.95) mL (*p* < 0.0001). Lesion volume significantly expanded in 37 of 61 measured lesions (60.7%), as defined by a longitudinal volume increase that exceeded inter-rater variability. Longitudinal analyses showed similar changes in lesion volume using the ground-truth lesion segmentations. Inter-scan duration was not associated with the magnitude of lesion growth. While the proposed tool requires further refinement and validation, we show that reliable and efficient semi-automated lesion segmentation is feasible in studies of chronic TBI, creating opportunities to elucidate mechanisms of post-traumatic neurodegeneration.

## 1 Introduction

Traumatic brain injury (TBI) is a well-established risk factor for neurodegenerative diseases (1). The pathophysiologic mechanisms that link TBI to post-traumatic neurodegeneration (PTND) are not fully understood, though emerging evidence implicates a “polypathology” (2) that includes axonal injury (3), tau deposition (4), vascular injury (5, 6), and neuroinflammation (3). An underexplored factor in the pathogenesis of PTND is the potential impact of focal cortical lesions, such as cerebral contusions, which are amongst the most common lesions in individuals with TBI (7). It is unknown whether focal lesion size evolves during the chronic stage of TBI (i.e., more than 1 year post-injury) and whether this may contribute to clinical decline.

In addition to a paucity of longitudinal studies in individuals with chronic TBI, a key barrier to elucidating the impact of cortical lesions on PTND pathogenesis is methodological. Historically, lesions that disrupt the surface of the cerebral cortex have prevented MRI segmentation tools from accurately differentiating lesion boundaries from the pial surface or the gray-white matter junction (8–11). Similarly, lesions that involve the white matter may lead to erroneous segmentation of lesion boundaries from nearby cortical and subcortical structures (12, 13). As a result, segmentation tools distributed with imaging analysis programs such as FreeSurfer (14), FSL (15), and SPM (16) have been unable to robustly measure longitudinal lesion growth. Hence, individuals with lesions have typically been excluded from TBI studies of cortical and subcortical volumetrics (17, 18). Achieving precise segmentations of lesions and nearby anatomic structures is essential for downstream workflows, including reconstruction of cortical surfaces and generation of volumetric measures (19), yet preliminary efforts to achieve this goal have required substantial time by operators trained in human neuroanatomy (11).

To address this methodological barrier and knowledge gap, we performed a longitudinal MRI study of individuals with chronic TBI and leveraged recent innovations in machine learning image analysis (20, 21) to create a semi-automated lesion segmentation tool. We tested the ability of this semi-automated lesion segmentation tool to detect longitudinal changes in lesion volume in individuals with chronic TBI enrolled in the Late Effects of TBI (LETBI) study (22). Our goal was to develop a tool that provides reliable and efficient measurement of cortical lesions to accelerate the study of PTND pathogenesis in individuals with chronic TBI.

## 2 Materials and methods

### 2.1 Participant selection

Between 2014 and 2023, 305 participants were enrolled in the ongoing LETBI study at Icahn School of Medicine at Mount Sinai (ISMMS) and the University of Washington (UW) (22). The LETBI study recruits individuals with a history of moderate or severe TBI. We used the United States Department of Defense classification of moderate TBI, which includes individuals considered by other classification systems as having “complicated mild” TBI (i.e., mild by Glasgow Coma Scale score criteria but with an intracranial lesion detected by brain imaging) (23). For the present longitudinal study, participants needed to have two MRI scans during consecutive study visits (≥2 years apart), each including T1-weighted (T1w) multi-echo magnetization prepared gradient-recalled echo (MEMPRAGE) scans (24) with a resolution of 1 mm isotropic.

Based on these criteria, 249 participants were excluded (*n* = 220 not yet eligible for second study visit, *n* = 29 without a T1w MEMPRAGE MRI dataset at both time points). Of the *n* = 220 excluded participants, scans from *n* = 10 were randomly selected to form the inter-rater dataset. Of the remaining 56 participants, at least one cortical lesion was identified in *n* = 24 (42.9%) by a trained rater who visually inspected the T1w images. Lesions were defined by visible disruptions in the cortical gray matter or cortical gray/white junction. Lesions could extend into the adjacent subcortical white matter.

### 2.2 Data acquisition, quality assessment, and processing

T1w images were obtained using Siemens Skyra, Philips Achieva, and Philips Ingenia Elition X scanners at 3 Tesla field strength. The images were acquired at 1 mm isotropic resolution. Siemens Skyra scans used a repetition time (TR) of 2,530 ms and echo times (TE) ranging from 1.79 ms to 7.37 ms. Philips Achieva scans used a TR of 2,530 ms and TEs ranging from 1.67 to 7.07 ms. Philips Ingenia Elition X scans used a TR of 2,530 ms and a TE of 2.14 ms. Further information about the number of scans obtained from each scanner is provided in Table 1. Eleven participants underwent imaging on one scanner for their initial scan and a different scanner for their follow-up scan due to upgrades occurring during the study follow-up periods. Additional sequence parameters for the T1w sequences on each scanner have been previously reported (22).

**Table 1:** MRI acquisition parameters for study participants.

**Manufacturer**	**Model**	**Field strength (*T*)**	**TR/TE (msec/msec)**	**Number of scans**
Siemens	Skyra	3T	2,530/1.79–7.37	18
Philips	Achieva	3T	2,530/1.67–7.07	11
Philips	Ingenia Elition X	3T	2,530/2.14	19

Qualitative and quantitative data quality assessments were performed on the processed images of all 24 subjects at both time points. Data uniformity and comparability across subjects and scanning platforms were examined, given the variations in sequence parameters. Visual quality assessments were based on the accuracy of FreeSurfer-generated surfaces (excluding those encompassing lesioned tissue) and the segmentation of subcortical structures, utilizing an accuracy rating scale adapted from Diamond et al. (11). Signal-to-noise ratio (SNR) and contrast-to-noise ratio (CNR) were measured using the FreeSurfer tools “wm-anat-snr” and “mri_cnr,” calculating SNR in white matter (WM) and the average of the WM-grey matter (GM) and GM-cerebrospinal fluid (CSF) contrasts, respectively. While no subjects were excluded due to quality assessment measures, differences were observed between the SNR distributions of enrollment sites, as reported in Table 2.

**Table 2:** Quantitative quality assessment of MRI data at each enrollment site.

**Enrollment site**	**Longitudinal cohort**	**SNR**	**CNR**
MSSM	*n =* 9	23.30 +/− 5.73	0.93 +/− 0.19
UW	*n =* 15	39.29 +/− 12.21	0.75 +/− 0.14

The T1w images were then processed, and the surfaces were constructed, using FreeSurfer v7.4 (14). FreeSurfer processing involves motion correction, averaging of T1w images, removal of non-brain tissue, automated Talairach transformation, and segmentation of brain structures. It also includes intensity normalization, GM/WM boundary tessellation, and topology correction. Further steps involve surface deformation, surface inflation, spherical atlas registration, cortical parcellation, and the creation of curvature and sulcal depth maps. To robustly segment neuroanatomic structures in brains with heterogeneous pathology, we used the Sequence Adaptive Multimodal SEGmentation (SAMSEG) tool (25, 26), instead of the default automated segmentation (aseg) tool, before FreeSurfer recon-all. FreeSurfer reconstructions for all participants were completed successfully.

### 2.3 “Ground truth” lesion segmentation

Ground truth segmentations for all participants were established through manual tracing performed by a neurologist blinded to subject identification and time point. The process involved loading each T1w image into the FreeSurfer image viewer, Freeview. A blank label volume was created using the same geometry as the T1w image. The neurologist (BLE) then manually segmented each lesioned area using the voxel edit tool, ensuring accurate and detailed delineation of the lesions. All segmentations were initially performed on a single label volume, which was later separated into unique values to indicate the presence of multiple lesions for individual subjects, thus creating a detailed ground truth segmentation volume at each time point for all participants.

### 2.4 Semi-automated lesion segmentation

**Figure 1:**
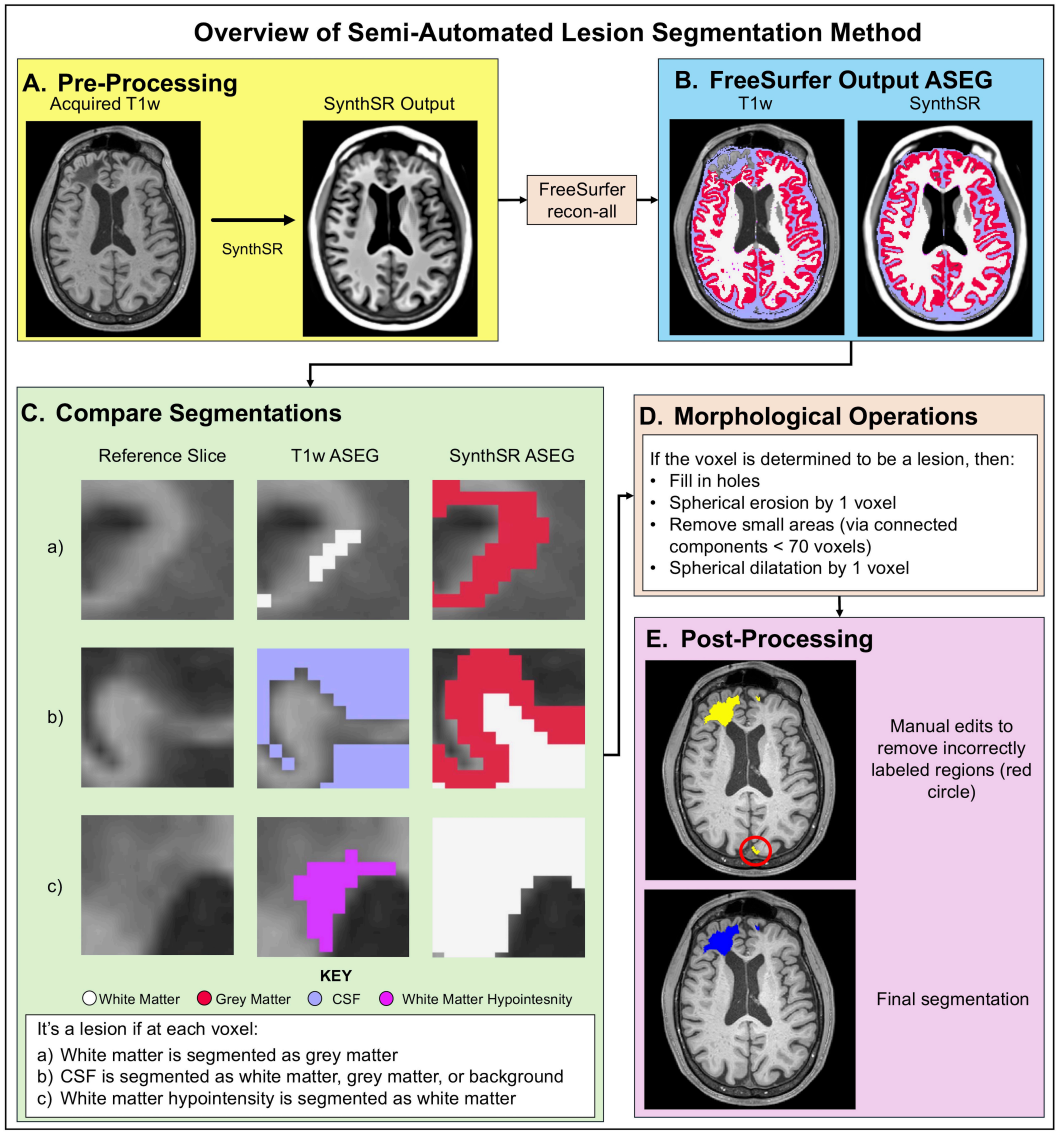
Overview of semi-automated lesion segmentation method. SynthSR images are generated for each acquired T1w image **(A)**. Both images are then processed through FreeSurfer recon-all, resulting in ASEG label volumes **(B)**. The SynthSR-ASEG is compared to the acquired T1w ASEG to highlight segmentation differences. Segmentation differences are identified voxel-by-voxel by comparing SynthSR ASEG and T1w ASEG volumes using predefined tissue-class rules: a voxel is classified as lesion if its label changes from white matter in ASEG to gray matter in SynthSR-ASEG, or from CSF to white matter, gray matter or background, or from white matter hypointensity to white matter **(C)**. Voxels meeting these criteria are classified as lesions and refined through morphological operations, including hole filling, erosion, small component removal, and dilation. **(D)**. Finally, the cleaned segmentation is reviewed for errors, including incorrectly labeled anatomy or missed lesions, and corrected to produce the final modified, semi-automated segmentation **(E)**.

To minimize time requirements and reduce false negatives (i.e., missed labeling) in manual tracing, we developed a novel method for semi-automated lesion segmentation intended to be implemented into existing FreeSurfer workflows. As illustrated in Figure 1A, we leveraged SynthSR (20, 21), a publicly available tool integrated within FreeSurfer that turns an MRI scan of any orientation, resolution, and contrast into a 1 mm isotropic T1w image while inpainting lesions.

We applied SynthSR to T1w images for all participants and then repeated the FreeSurfer recon-all process on the synthesized images (Figure 1B). We defined lesional voxels by comparing the SAMSEG (27) labels from the synthesized image with those from the original T1w image (Figure 1C) using the following rules: a voxel is defined as a lesion if the segmentation label changed: (1) from white matter (in the original T1w recon) to gray matter (in the SynthSR recon); or (2) from CSF to background/white matter/gray matter; or (3) from white matter hypo-intensity to white matter. These rules were determined heuristically based on the segmentation label changes inside the lesional areas from a subset of our sample (*n* = 5, randomly selected from the entire cohort and blinded to time point). Additional details and FreeSurfer segmentation class information is outlined in Table 3.

**Table 3:** Segmentation rules to identify lesional voxels.

**T1 SAMSEG ASEG (FreeSurfer Label ID)**	**SynthSR ASEG (FreeSurfer Label ID)**
White matter (2, 41)	Gray matter (3, 42)
CSF (24)	White matter (2, 41)
CSF (24)	Gray matter (3, 42)
CSF (24)	Background (0)
White matter hypointensity (77)	White matter (2, 41)

Additional details regarding FreeSurfer labels are provided at https://surfer.nmr.mgh.harvard.edu/fswiki/FsTutorial/AnatomicalROI/FreeSurferColorLUT.

To determine the segmentation rules, we relied on the label changes within the ground truth lesion masks from those 5 subjects. Specifically, within the lesional areas defined by the ground truth masks, we counted the number of voxels that had any changes in their labels between the original SAMSEG and the SynthSR SAMSEG, and we then sorted them from the largest to the smallest. We used the label change with the largest voxel count as our first rule and evaluated the Dice scores across the entire image against the ground truth. We then used a “greedy approach” by adding the label change with the second, third, etc. largest voxel counts and re-evaluated the Dice. We stopped this rule selection process when the Dice score started decreasing.

Subsequently, we applied morphological image processing (28) to remove false positives, reduce noise, and ensure that the detected lesional areas are topologically correct, including hole filling, spherical erosion/dilation, and area opening (Figure 1D). Successful application of this pipeline facilitated the identification of clusters of lesioned voxels in the SynthSR inpainted volume, yielding an initial automated lesion segmentation mask (Figure 1E). A trained rater then performed manual edits (the only manual step in the semi-automated lesion segmentation method) to enhance the accuracy of lesion segmentation boundaries, yielding a final semi-automated lesion mask. In post-processing, this mask was separated into unique values to identify multiple lesions for a single subject. The rater performing manual edits for the semi-automated lesion segmentation method was blinded to the “ground truth” manual segmentations performed by the prior rater.

### 2.5 Evaluation of inter-rater variability for the manual editing step of the semi-automated method

To determine inter-rater variability for the manual editing step of the semi-automated lesion segmentation method, we randomly selected 10 T1w images with lesions from the LETBI dataset that were not included in the 24-subject longitudinal dataset (i.e., subjects for whom longitudinal data were not yet available). These 10 independent T1w images were edited by three raters, each of whom traced every lesion present on each scan. Raters were provided with SynthSR-generated segmentation masks (i.e., the initial automated lesion mask, as represented by the yellow lesion mask in Figure 1E) and instructed to revise the segmentations, creating a final semi-automated lesion mask (as represented by the blue lesion mask in Figure 1E). In post-processing, this mask was further separated into unique values to identify multiple lesions for individual subjects. The raters' final lesion masks were then compared to measure inter-rater variability.

To test inter-rater variability, we performed Bland-Altman analyses for each pair of raters (Rater 1 vs. Rater 2, Rater 1 vs. Rater 3, and Rater 2 vs. Rater 3), calculating the mean difference (bias) and 95% limits of agreement (LoA). This analysis was completed on the volume of each lesion, with multiple lesions from patients contributing to the analysis and each lesion being treated as an independent observation. Additionally, we computed the Intraclass Correlation Coefficient (ICC) to assess the reliability of lesion-tracing. These analyses together assessed both agreement and reliability in lesion volumes, identifying any systematic biases or random variability. The LoA established a benchmark for subsequent statistical testing of longitudinal lesion expansion, allowing us to determine whether observed changes in lesion volume over time reflect lesion expansion or variability in the method.

### 2.6 Quantifying the contribution of the initial segmentation to the semi-automated method

We quantified the contribution of the initial, fully automated segmentation step and the degree of subsequent manual editing needed to generate the final semi-automated segmentations. For this analysis, we calculated Dice coefficients for each lesion against the ground truth before and after manual refinement. We assessed whether these differences were significantly different from zero using Wilcoxon signed-rank tests. Bland–Altman plots were used to assess agreement between the two approaches by plotting the lesion-wise difference in Dice against the lesion-wise mean Dice.

For the voxel-wise comparisons, we calculated voxel-level true positives (TP), false negatives (FN), true negatives (TN), and false positives (FP) by comparing ground truth with both initial segmentation and refined semi-automated segmentations at corresponding voxel locations. TP were voxels where both ground truth and segmentation were non-zero; FP were voxels where the segmentation was non-zero, but the ground truth was zero; TN were voxels where both were zero; and FN were voxels where the ground truth was non-zero, but the segmentation was zero. For each lesion, we then derived the Dice coefficient [2 TP/(2 TP + FP + FN)], sensitivity [TP/(TP + FN)], specificity [TN/(TN + FP)], and precision [TP/(TP + FP)]. Given that zero-valued background voxels were highly prevalent, we emphasize precision rather than specificity, as it more directly reflects the reliability of positive voxel detections. Per-lesion metrics were then aggregated across subjects by reporting the mean and standard deviation for each comparison.

We next evaluated computational efficiency by measuring the total time required for recon-all completion. Runtimes for the SAMSEG and SynthSR workflows were compared with those from a standard FreeSurfer v8.0 run to assess potential time savings.

Lastly, we quantified manual workload using an edited fraction metric, calculated as (deleted + added) voxels divided by the final semi-automated lesion voxels. This reflects the proportion of voxels requiring change during manual refinement, providing an estimate of editing effort.

### 2.7 Comparison of automated, semi-automated and ground truth segmentations

**Figure 2:**
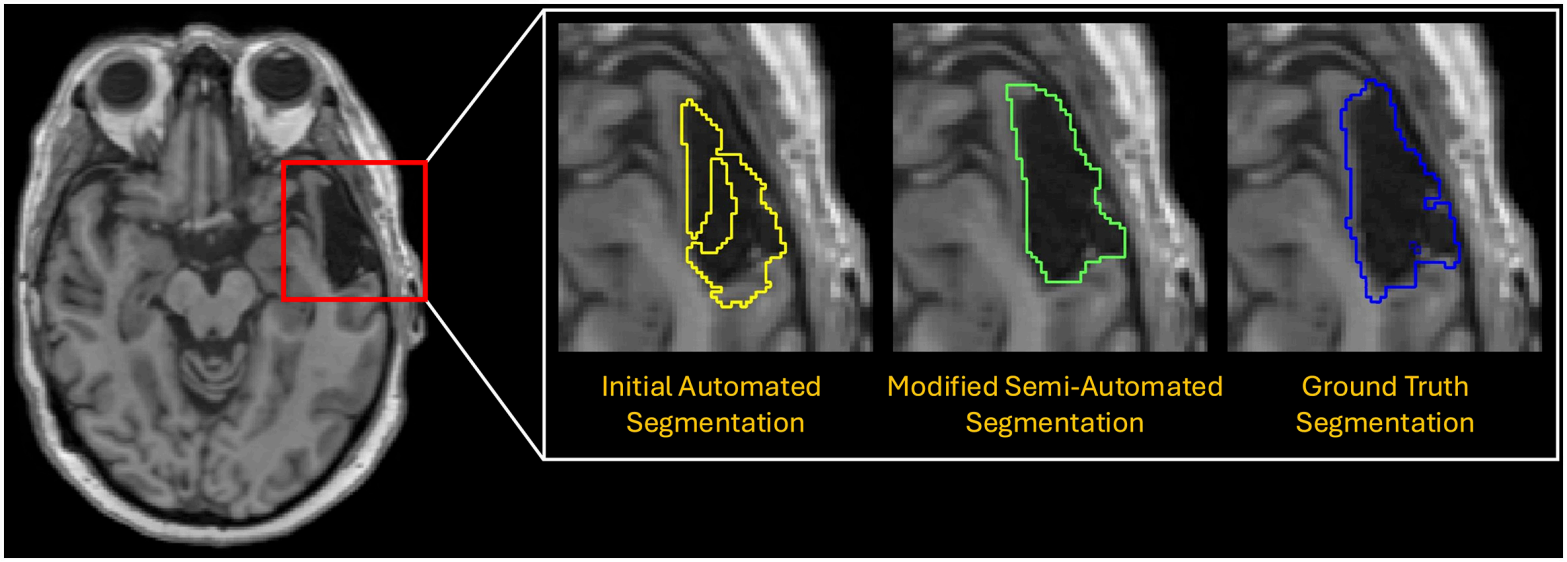
Comparison of the initial automated lesion segmentation (yellow), the modified semi-automated segmentation (green), which was revised by a trained rater, and the manually traced “ground truth” segmentation (blue).

To evaluate the agreement of methods, we compared edited semi-automated segmentations to the ground-truth segmentations (Figure 2) at both time points using Wilcoxon signed-rank tests and Bland-Altman analyses. The Wilcoxon tests assessed whether there were statistically significant differences in the volumes generated by the two methods, while the Bland-Altman analyses estimated the mean difference (bias), standard deviation, and limits of agreement between them.

To complement the volumetric comparisons, we performed voxel-wise comparisons of the initial automated segmentations and the semi-automated segmentations against the ground truth segmentations. By comparing both automated and semi-automated segmentations against the ground truth segmentations, we provide a quantitative measure of the changes in Dice scores, sensitivity, and precision that occur during the manual refinement process of semi-automated segmentation.

### 2.8 Testing for longitudinal changes in lesion volume

**Figure 3:**
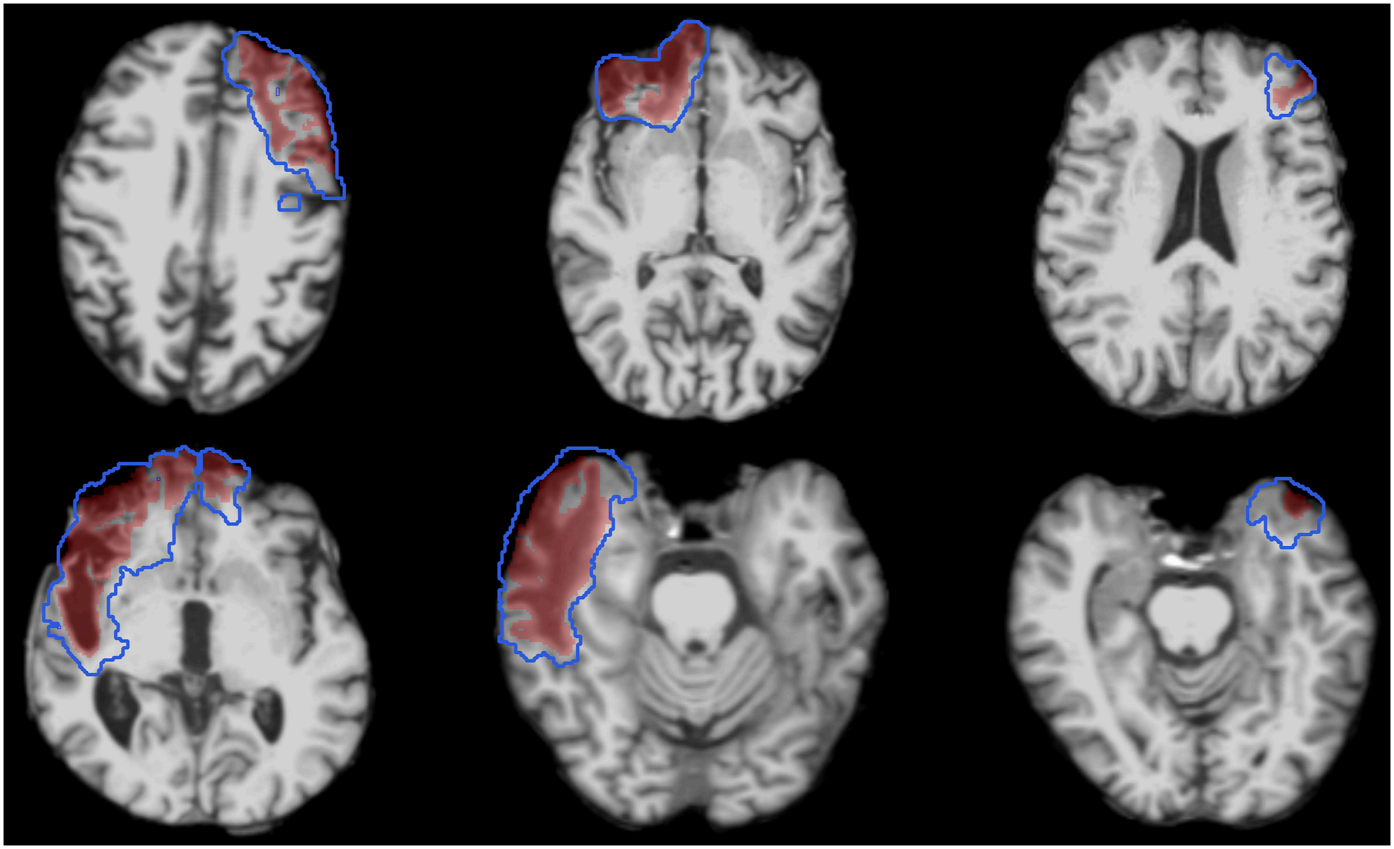
T1w images from Visit 1 of six representative subjects demonstrating the heterogeneous nature of lesion size and location. Images are overlayed with the ground truth lesion segmentations from Visit 1 (red shaded regions) and Visit 2 (blue outlined regions).

We hypothesized that there are detectable changes in lesion volume when comparing Visit 1 to Visit 2 for the entire cohort and when comparing single-subject changes in lesion volume to the null distribution of inter-rater variability (Figure 3). We tested these two hypotheses using the semi-automated segmentations, as well as the ground truth segmentations. We began by comparing lesion voxel volumes, measured in mL, between Visit 1 and Visit 2, and then calculating the difference (Visit 2 – Visit 1) for each pair of measurements to determine the change in lesion volume. The statistical significance of these changes was assessed using the Wilcoxon signed rank test.

### 2.9 Testing for changes in lesion volume compared to inter-rater variability

Next, we tested whether the observed longitudinal changes in Dice overlap and lesion volume exceeds the degree of inter-rater variability. Dice overlap and lesion volume differences were calculated for each lesion segmentation. For inter-rater data, Dice overlap and lesion volume differences were averaged across the three raters to generate composite scores for each lesion. For the semi-automated and ground truth groups, Dice overlap and lesion volume differences were derived by comparing Visit 2 to Visit 1 segmentations. Statistical significance of the differences between longitudinal changes in lesion measurements (i.e., Dice overlap and volume) and inter-rater variability was assessed using the Wilcoxon rank sum test, with a significance threshold of 0.05, to account for outliers and the small sample size. Finally, we operationally defined lesion expansion at the individual lesion level based on an increase in lesion volume greater than 2 times the SD over the mean of the inter-rater volume variability, consistent with prior neuroimaging studies (29). In a secondary analysis, we tested for lesion expansion using a 1.5 SD cutoff based on the application of this statistical threshold to define abnormal cognitive performance in clinical practice (30).

Longitudinal analyses of lesion volumes were performed in the subject's native space at each time point. This method was selected instead of using the FreeSurfer longitudinal pipeline, which combines the two time points to generate a base image (31). The averaging process in the FreeSurfer pipeline would obscure the examination of lesion progression by blending the time points together, thus failing to capture dynamic changes in lesion size and location. By performing analyses in native space, we maintain the integrity of individual time point data, allowing for precise tracking of lesion growth and development over the study period without introducing registration artifacts.

### 2.10 Evaluation of factors associated with lesion volume change

We examined the relationship between changes in lesion size (measured in mL) and the interval between imaging visits (measured in days). For changes in lesion size, we use the values previously calculated for analyses as described in Section 2.8. Individual lesion clusters were matched between visits to directly compare changes over time. Pearson correlation coefficient (*R*) and two-tailed *p*-value were computed to assess the strength and significance of any linear relationship between changes in lesion size and duration between study visits. We applied Ordinary Least Squares (OLS) regression analysis to further investigate how age, sex and interval between study visits relate to changes in lesion volume.

In addition, we examined whether voxel intensity changes introduced by SynthSR influenced lesion volume differences or the extent of manual editing. For each visit and lesion, we quantified the number and volume of lesioned white matter voxels that underwent a signal change after SynthSR processing. These measures were compared with the manual-edit overlap and absolute lesion volume difference using visit-wise Spearman correlations.

## 3 Results

### 3.1 Participant and lesion characteristics

**Figure 4:**
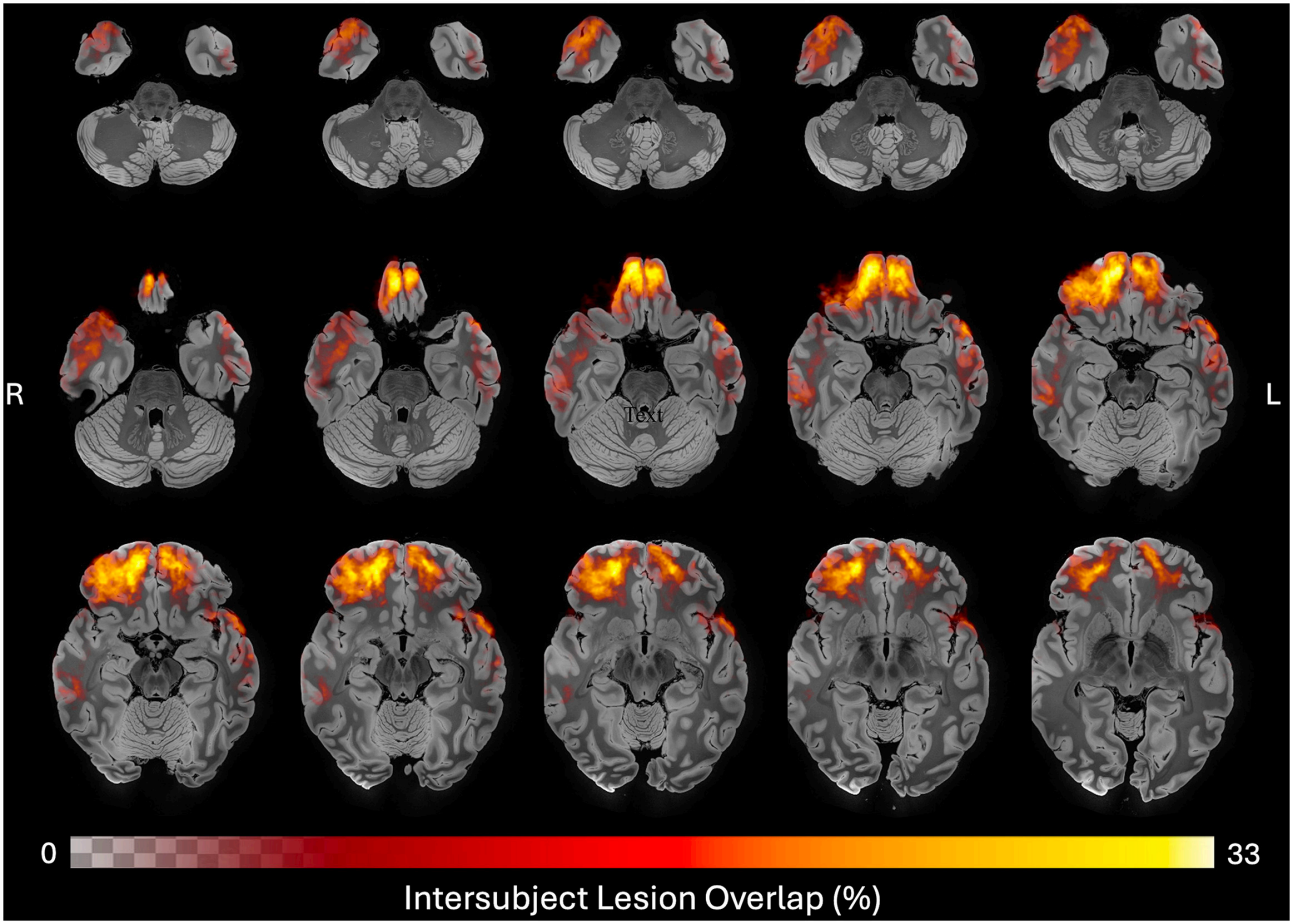
Neuroanatomic distribution of ground truth lesions across time points. Heatmap of all 48 ground truth lesion tracings registered to MNI space and overlayed on a 100 micron MRI template (46), revealing a predominance of frontotemporal cortical lesions in this cohort. Color and opacity of the heatmap are modulated by the percent of lesion traces in each voxel, with the maximum overlap observed being 33%.

The 24 longitudinal participants ranged in age at Visit 1 from 33 to 73 years old, with a median age of 55.8 years (IQR = 14.3). Of these participants, nineteen were males. The 10 inter-rater participants ranged in age from 31 to 73 with a median age of 51.8 years (IQR = 22.8). Nine of the inter-rater participants were male. Additional descriptive statistics are provided in Table 4. Lesions were heterogeneous with respect to their neuroanatomic locations and were most prevalent in the anterior frontal and temporal lobes (Figure 4). For the 24 individuals studied here the ground truth lesion volume at Visit 1 ranged from 0.47 to 53.28 mL (median = 8.81 mL, IQR = 12.21 mL) and Visit 2 lesion volume ranged from 0.99 to 122.52 mL (median = 19.29 mL, IQR = 34.42 mL). For the 10 different subjects in inter-rater cohort, the lesion volume ranged from 0.93 to 29.38 mL (median = 4.44 mL, IQR = 7.53 mL).

**Table 4:** Patient and lesion characteristics.

**Characteristics**	**Longitudinal dataset (*****n =*** **24)**			**Inter-rater dataset (*****n =*** **10)**		
	***M***	**SD**	**Range**	***M***	**SD**	**Range**
Age at first visit (years)	55.8	11.2	33–73	51.8	14.5	31–73
Visit interval (days)	1,328.5	498.7	734–2366	N/A	N/A	N/A
Sex (male:female)	19:5	N/A	N/A	9:1	N/A	N/A
Lesion volume (visit 1, mL)	N/A	N/A	0.47–53.28	N/A	N/A	0.93–29.38

### 3.2 Inter-rater variability

We first assessed agreement among the three raters using Bland-Altman analysis (Table 5). The mean bias values between each pair of raters ranged from −0.98 mL to 1.44 mL, with LoA spanning from −3.57 mL to 4.60 mL. Additionally, the ICC was calculated to evaluate reliability among raters. The ICC (2,1) was 0.98 (95% CI: 0.93–0.99), indicating excellent reliability. These results demonstrate a high level of consistency across the raters, with minor differences likely attributable to individual rater preferences or the inherent complexity of lesion-tracing in chronic TBI. Variability within the LoA reflects the heterogenous and complex nature of the lesions, leading to differing identifications and tracings by raters.

**Table 5:** Inter-rater Bland Altman agreement results.

**Comparison**	**Mean difference (bias) [mL]**	**Dice (mean ±SD)**	**Upper LOA [mL]**	**Lower LOA [mL]**
Rater 1 vs. Rater 2	1.44	0.82 ± 0.07	4.60	−1.72
Rater 1 vs. Rater 3	0.47	0.75 ± 0.18	2.03	−1.10
Rater 2 vs. Rater 3	−0.98	0.77 ± 0.17	1.62	−3.57

### 3.3 Contribution of the initial automated segmentation to the semi-automated pipeline

**Figure 5:**
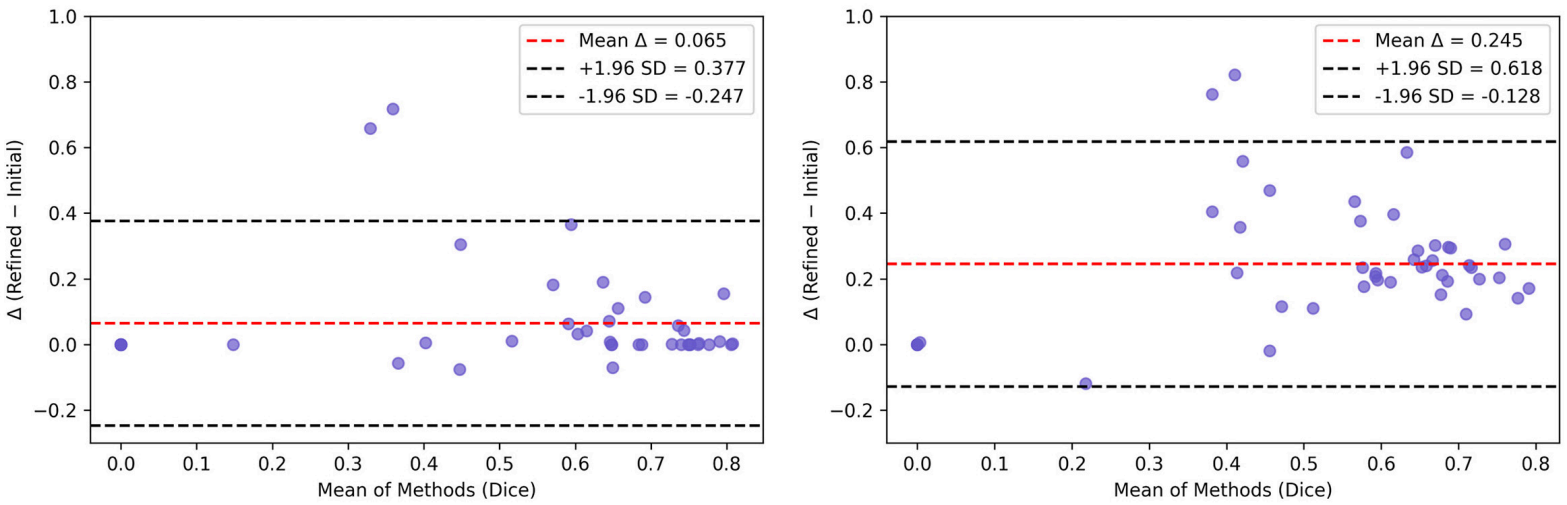
Bland–Altman plots comparing initial vs. refined semi-automated Dice (against ground truth) per lesion for Visit 1 **(left)** and Visit 2 **(right)**.

Wilcoxon signed-rank tests showed that Dice coefficients significantly increased after refinements at both visits (Visit 1: *W* = 49.0, *p* < 0.0008; Visit 2: *W* = 8.0, *p* < 0.0001). Bland-Altman analyses indicated mean Dice improvements of +0.065 at Visit 1 (95% LoA: −0.247 to 0.377) and +0.245 at Visit 2 (−0.136 to 0.615) (Figure 5). These results demonstrate that manual refinement recovers, on average, an additional 6.5 % and 23.9 % Dice overlap in Visit 1 and 2, respectively.

Table 6 details the improvement in Dice score and sensitivity achieved by the semi-automated segmentations, as compared to the initial automated segmentations, at each visit. At Visit 1, Dice increased from 0.47 to 0.54 and sensitivity from 0.45 to 0.56, while precision decreased slightly from 0.72 to 0.70. At Visit 2, Dice improved from 0.40 to 0.64 and sensitivity from 0.29 to 0.63, with precision dropping from 0.87 to 0.77. These observations suggests that the manual refinement step of the semi-automated pipeline increases volumetric overlap with the ground truth segmentation and recovers more true lesion voxels than the initial segmentation captures alone. The observed increase in Dice and sensitivity paired with the decrease in precision at both timepoints indicates that while more true lesion voxels are being captured, some non-lesion voxels are also being added to the segmentation masks during the manual refinement.

**Table 6:** Group-level performance metrics against ground truth segmentations for initial vs. semi-automated segmentations.

**Segmentation methods**	**Visit 1**				**Visit 2**			
	**Dice (*****M*** ±**SD)**	**Sensitivity (*****M*** ±**SD)**	**Precision (*****M*** ±**SD)**	**Specificity (*****M*** ±**SD)**	**Dice (*****M*** ±**SD)**	**Sensitivity (*****M*** ±**SD)**	**Precision (*****M*** ±**SD)**	**Specificity (*****M*** ±**SD)**
Initial automated segmentation	0.47 ± 0.30	0.45 ± 0.30	0.72 ± 0.17	1.00 ± 0.00	0.40 ± 0.23	0.29 ± 0.17	0.87 ± 0.21	1.00 ± 0.00
Semi-automated segmentation	0.54 ± 0.30	0.56 ± 0.31	0.70 ± 0.71	1.00 ± 0.00	0.64 ± 0.28	0.63 ± 0.28	0.77 ± 0.22	1.00 ± 0.00

We assessed computational efficiency by extracting recon-all completion times from log files for SAMSEG, SynthSR, and FreeSurfer v8. Across all participants and visits, runtimes were shorter with SynthSR (2.43 ± 0.40 h; 2.41 ± 0.43 h) and SAMSEG (2.67 ± 0.42 h; 2.75 ± 0.42 h) than with FreeSurfer v8 (3.88 ± 0.76 h; 3.84 ± 0.46 h), representing an average ~1.4 h reduction and lower variability (Table 7).

**Table 7:** Recon-all runtimes by method and visit.

**Visit**	**SAMSEG**		**SynthSR**		**FreeSurfer v8**	
	**M** ±**SD [HH:MM]**	**Min–Max**	**M** ±**SD [HH:MM]**	**Min–Max**	**M** ±**SD [HH:MM]**	**Min–Max**
Visit 1	2.67 ± 0.42	2.02–3.64	2.43 ± 0.40	1.80–3.34	3.88 ± 0.76	3.05–6.61
Visit 2	2.75 ± 0.42	1.86–3.65	2.41 ± 0.43	1.90–3.35	3.84 ± 0.46	3.13–4.62

Although editing time was not recorded, the edited fraction was small at Visit 1 (median = 0.11, IQR = 0.00–0.43) and larger at Visit 2 (median = 0.59, IQR = 0.51–0.71), indicating that refinement typically required labeling substantially fewer voxels than fully manual tracing. Together with the observed Dice improvements after refinement, these findings suggest reduced manual effort despite some increases in non-lesion voxels. The time required for each segmentation method varied depending on lesion burden. We estimated that lesion adjustment using the semi-automated method required approximately 10–20 min per scan, compared to 60–90 min for manual segmentation.

### 3.4 Semi-automated segmentation performance compared to ground truth segmentation

At Visit 1, the semi-automatic volume measurements yielded a mean bias of 2.42 mL (SD = 7.06 mL, median = −0.01 mL, IQR = 6.36 mL) relative to the ground truth measurements. The limits of agreement ranged from −11.41 mL to 16.25 mL, demonstrating a reasonably close alignment between the two methods. The Wilcoxon signed-rank test indicated no statistically significant difference (*W* = 276.00, *p* = 0.26), suggesting that the semi-automated method closely approximates the ground truth.

At Visit 2, the semi-automated measurements showed a higher mean bias of −3.08 mL (SD = 9.71 mL, median = −2.49 mL, IQR = 5.99 mL) compared to the ground truth, with a wider range of agreement (−22.13 mL to 15.96 mL). This observation suggests that measurement differences varied more than in the first visit. A Wilcoxon signed-rank test revealed a significant difference between the two methods at this timepoint (*W* = 197.00, *p* = 0.019), suggesting that the discrepancies between the semi-automated and ground truth measurements were more pronounced at this visit.

**Figure 6:**
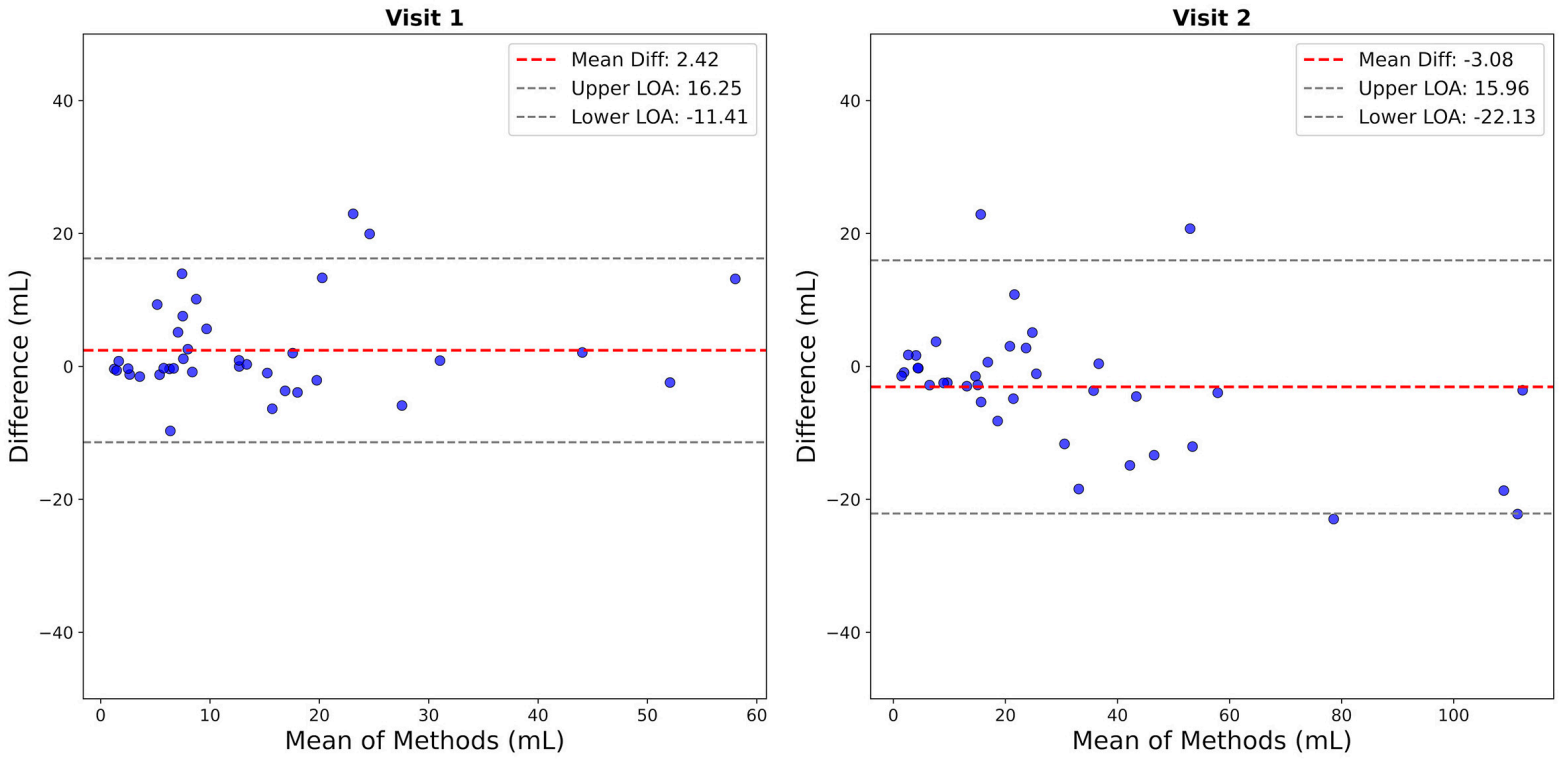
Bland-Altman agreement plots comparing lesion volume measurements across visits and methods. Each plot shows the difference vs. the mean, with bias (dashed red lines) and limits of agreement (dashed black lines).

Collectively, these findings at Visit 1 and Visit 2 suggest that the semi-automated method provides a reliable alternative to ground truth tracing, offering comparable accuracy and consistency, despite increased variability at the second timepoint (Figure 6).

### 3.5 Longitudinal changes in lesion volume

Longitudinal changes in lesion sizes derived from semi-automated segmentations at Visit 1 and Visit 2 ranged from −0.11 to 55.21 mL. The Wilcoxon signed-rank test results yielded a statistic of 1.0 at *p* < 0.0001, indicating an increase in lesion volume between Visit 1 and Visit 2. Repeating the Wilcoxon signed-rank test using the ground truth segmentations similarly revealed an increase in lesion volume, ranging from 1.30 to 79.45 mL (*W* = 1.00, *p* < 0.0001).

### 3.6 Changes in lesion volume compared to inter-rater variability

**Figure 7:**
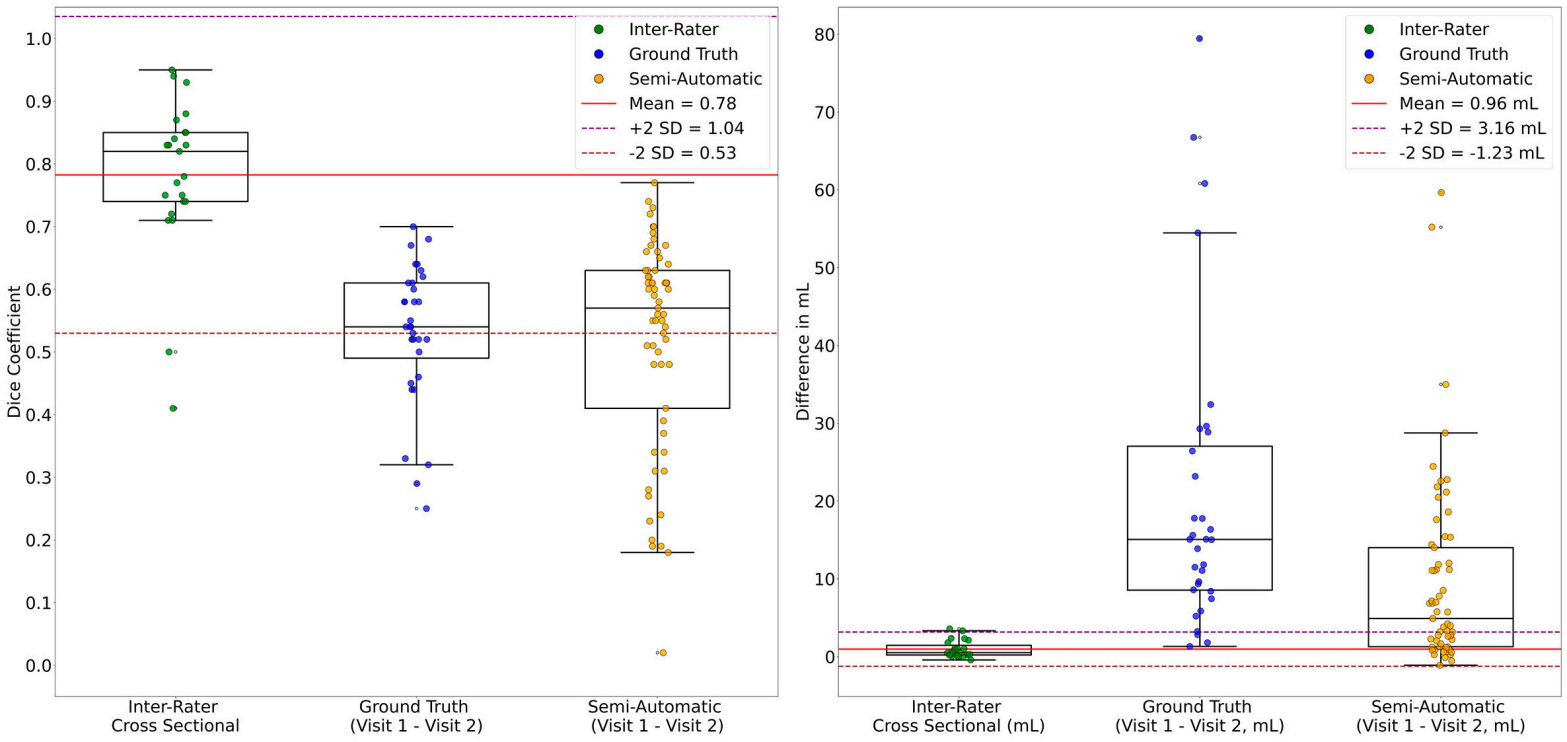
Comparison of Longitudinal Lesion Changes with Inter-rater Variability. The longitudinal changes in Dice scores (**left panel**) and volume measurements (**right panel**) from Visit 1 to Visit 2 are compared to their respective inter-rater reliability measures. The respective mean (**red line**) and 2 SD intervals (**purple dashed lines**) pertain to the inter-rater data.

The longitudinal changes in Dice overlap from Visit 1 to Visit 2 exceeded inter-rater variability for both the semi-automated method (*W* = 1299.0, *p* < 0.0001) and the ground truth method (*W* = 684.5, *p* < 0.0001). Similarly, the increase in lesion volume from Visit 1 to Visit 2 exceeded inter-rater variability (i.e., the volume difference between raters for the same lesion) for both the semi-automated method (*W* = 225.0, *p* < 0.0001) and the ground truth method (W= 16.0, *p* < 0.0001) (Figure 7). Further, 90.6% of lesions for the ground truth method and 60.7% of lesions for the semi-automated method experienced an increase in lesion volume greater than 2 times the SD over the mean of the inter-rater results (top dashed line in Figure 7, right). When using a threshold of 1.5 times the SD over the mean of the inter-rater results, 93.8% of lesions for the ground truth and 65.6% of lesions for the semi-automated experienced an increase in lesion volume.

### 3.7 Lesion size variation across different visit intervals

**Figure 8:**
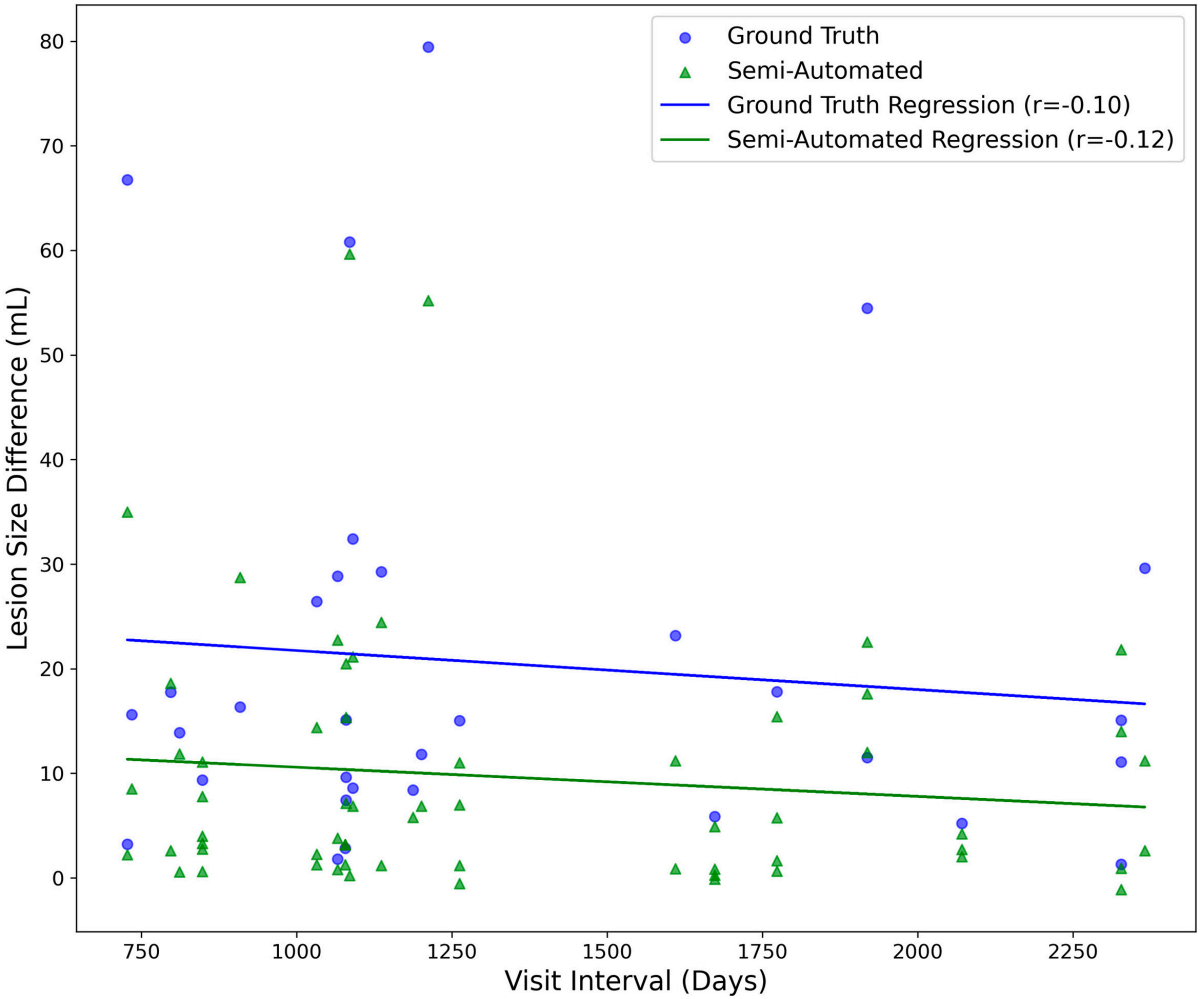
Correlation analysis examining the time between study visits and the calculated difference in lesion volume both for ground truth (**blue**) and semi-automated (**green**) methods.

Inter-visit intervals ranged from 727 to 2,366 days. Correlation analyses revealed no relationship between visit intervals and changes in lesion size for both the semi-automated (*R* = −0.12, *p* = 0.38) and ground truth (*R* = −0.10, *p* = 0.58) methods (Figure 8). Regression models accounting for age, sex, and visit interval explained only 7.3% (*R*^2^ = 0.073) and 5.2% (*R*^2^ = 0.052) of the variance in lesion volume change, for the semi-automated and ground truth methods, respectively. After adjusting for the number of predictors, the adjusted R^2^ values were 0.024 for semi-automated and −0.049 for ground truth, indicating minimal explanatory power. None of the individual predictors were statistically significant in either model (all p > 0.11). The overall F-statistics were 1.49 (*p* = 0.23) for semi-automated and 0.52 (*p* = 0.68) for ground truth, suggesting that the models did not effectively predict lesion volume changes. Together, these findings indicate that the observed changes in lesion size after TBI are not explained by age-related factors or influenced by sex, regardless of the measurement method used.

### 3.8 Impact of SynthSR-induced voxel intensity changes on lesion volume and editing burden

We further assessed whether SynthSR-induced voxel intensity changes were associated with lesion volume differences or editing burden (Table 8). Correlations between intensity change volume and lesion volume difference were small and non-significant (Visit 1: ρ = −0.26, *p* = 0.215, *q* = 0.287; Visit 2: ρ = 0.08, *p* = 0.713, *q* = 0.713). The intensity change count showed modest, visit-dependent correlations with manual-edit overlap (Visit 1: ρ = 0.38, *p* = 0.067, *q* = 0.134; Visit 2: ρ = 0.41, *p* = 0.047, *q* = 0.134), suggesting a potential but limited influence on manual editing requirements.

**Table 8:** Correlations between lesion intensity changes and manual-editing burden across visits.

**Association**	**Visit 1**			**Visit 2**		
	ρ	***p***	***q***	ρ	***p***	***q***
Absolute lesion volume difference (mL) ↔ Intensity-change volume within lesions (mL)	−0.263	0.215	0.287	0.079	0.713	0.713
Intensity-change voxels within lesions (count) ↔ Manual-edit overlap (count)	0.380	0.067	0.134	0.409	0.047	0.134

## 4 Discussion

In this longitudinal MRI study of 24 individuals with chronic TBI, we demonstrate the reliability and efficiency of a semi-automated cortical lesion segmentation tool. Our findings indicate that this semi-automated method performs robustly against ground-truth manual tracings to segment lesions with improved precision over an automated technique and with improved time efficiency compared to previously developed methods (11). Further, in a proof-of-principle application of the semi-automated lesion segmentation tool, we provide preliminary evidence that cortical lesions expand beyond 1 year post-injury, with 37 of 61 measured lesions (60.7%) expanding on MRI scans performed at least 2 years apart. These observations raise the possibility that lesion expansion may contribute to PTND—a finding that will require confirmation in larger longitudinal studies with clinical-radiologic-pathological correlations. The semi-automated lesion tool thus creates new opportunities to investigate the role of cortical lesions in the pathogenesis of PTND.

The semi-automated lesion segmentation tool developed here builds upon recent innovations in machine learning-based imaging analysis, most notably SynthSR (20, 21). What distinguishes this tool from previously developed lesion segmentation methods is: (1) increased efficiency when compared to traditional manual tracing; (2) compatibility with standard FreeSurfer outputs with a fully specified, scriptable sequence of steps to support reproducibility within FreeSurfer-based workflows; and (3) scalability to large datasets with anatomically guided segmentation. The new semi-automated tool demonstrates robust performance characteristics against “ground-truth” manual lesion segmentations, as evidenced by the strong agreement observed between the two methods at Visit 1. The observed discrepancies at Visit 2 may reflect a heightened sensitivity of the semi-automated method to more complex lesion morphologies, which could account for the increased variability in measurements. Collectively, these findings underscore the reliable performance of semi-automated segmentation compared to traditional manual tracing, but also the potential for future improvement in the reliability of the method.

Importantly, the semi-automated lesion segmentation tool requires human input to refine and optimize lesion boundaries—a step that reflects the inherent challenge of training automated tools to detect traumatic lesions, which often have heterogeneous signal characteristics related to variable distributions of gliosis, demyelination, and encephalomalacia. This manual editing step required approximately 10–20 min per MRI scan, and we estimate that several hours of training were required for each rater performing the manual editing. Nonetheless, the time required for the manual editing step in the newly proposed method is far less than for our previously published lesion segmentation method (11). While the prior tool required manual creation of set points along the entire lesion surface, the new method requires only a small number of voxel-based edits in volumetric space. A key future direction will be to determine whether full automation is reliable. This goal may be attainable via integration with recently developed methods such as VoxelPrompt (32) and FastSurfer-LIT (33). We currently recommend manual editing of segmented lesions until further studies confirm the reliability of fully automated methods.

The lesion expansion observed in this cohort is consistent with and builds upon the growing evidence base indicating that pathological processes in TBI persist and progress in the chronic setting, even beyond one year post-injury. The clinical significance of lesion expansion is well established in the acute stages of care (34), wherein expansion of an acute lesion may cause mass effect and herniation. In the chronic stage, there are many unanswered questions about the clinical relevance, underlying mechanisms, and temporal dynamics of TBI lesion expansion. Prior evidence from histopathology (3, 35) and neuroimaging studies suggests that inflammation persists in the chronic stage of TBI (36, 37), but whether chronic lesion expansion is attributable to inflammation, gliosis, microvascular ischemia, or some combination of factors is unknown. Elucidating mechanisms of chronic lesion expansion will require pathological-radiologic correlation analyses, which the LETBI study is designed to perform, given the premortem consent for autopsy provided by LETBI participants (22). The absence of an association between lesion expansion and time between scans suggests that lesion expansion occurs at variable rates, though this preliminary observation will require future studies with larger sample sizes to confirm.

Several limitations should be considered when interpreting the results of this study. The small sample size of 24 individuals with chronic TBI limits the generalizability of our results, necessitating larger cohorts for validation. Only investigating two imaging time points and the relatively brief follow-up period of 3.5 +/− 1.2 years is also insufficient to elucidate the long-term trajectory of lesion expansion and its implications for PTND. The potential contribution of cortical lesion expansion to the pathogenesis of PTND is unknown and will require future studies with sufficiently large sample sizes to account for other risk factors, as well as protective factors.

While the semi-automated tool improves efficiency, it still requires manual input for refining lesion boundaries, introducing potential variability and subjectivity. Additionally, the heterogeneous neuroanatomic locations and signal characteristics of traumatic lesions further complicate the segmentation process, as the tool may not uniformly handle all types of lesions with the same accuracy. It is currently unknown whether the semi-automated method detects lesions that evade detection by manual raters who do not have access to the initial automated lesion masks. Lastly, this study did not test for cognitive and functional correlates of lesion expansion—a crucial area for future research. Addressing these limitations will be essential for advancing our understanding of cortical lesion dynamics in chronic TBI.

A key goal in future studies will be to evaluate the accuracy and efficiency of the proposed semi-automated lesion segmentation technique in comparison to fully automated techniques that are being generated in the rapidly evolving field of deep learning. There is an increasing number of MRI segmentation algorithms that are being applied to a broad spectrum of neuropsychiatric diseases using deep learning tools (38–41), as well as applications in more widely available CT scans (42–44). Several such techniques have been applied to individuals with TBI (42, 45), underscoring the potential for fully automated deep learning techniques to supplement, or ultimately replace, the type of semi-automated lesion segmentation tool developed here.

Nevertheless, there are several potential benefits of a technique that leverages a robust software package like FreeSurfer, which has been intensively tested, verified and can handle a broad spectrum of neuroimaging data. Specifically, our technique obviates the need for extensive model training for different types of lesions, because it generates preliminary segmentations via a simple, reproducible pipeline amenable to subsequent curation. Standalone, dataset-specific deep learning approaches, by comparison, require further validation of their generalizability, entail maintenance and support, investment in replication infrastructure, and significant computational resources for training on new datasets. Also, because our approach relies on standard FreeSurfer outputs, it is immediately deployable and likely more feasible to integrate into existing neuroimaging pipelines than establishing a computational environment for more sophisticated automated segmentation.

## 5 Conclusions

In summary, we developed and implemented a semi-automated lesion detection tool that accurately identifies and efficiently quantifies the volume of cortical lesions in individuals with chronic TBI. Further, we provide proof-of-principle evidence that this lesion segmentation tool can detect longitudinal lesion growth in the chronic stage of TBI. Future applications of this tool have the potential to elucidate the pathophysiologic links between lesion expansion and the clinical expression of PTND, including in individuals with TBI resulting in large cortical lesions that would otherwise exclude them from analyses of neuroimaging data. Ultimately, the integration of lesion segmentation into clinical MRI workflows has the potential to inform preventive, diagnostic, prognostic, and therapeutic strategies for individuals with chronic TBI.

## Data Availability

The analytic code used in this study is available at https://github.com/ComaRecoveryLab/semiauto-cortical-lesion-expansion. The source data is available upon reasonable request to the corresponding author.

## References

[1] Dams-O'connor K Ketchum JM Cuthbert JP Corrigan JD Hammond FM Haarbauer-Krupa J. Functional outcome trajectories following inpatient rehabilitation for TBI in the United States: a NIDILRR TBIMS and CDC interagency collaboration. J Head Trauma Rehabil. (2020) 35:127–39. 10.1097/HTR.0000000000000484 31033744 PMC6814509

[2] Kenney K Iacono D Edlow BL Katz DI Diaz-Arrastia R Dams-O'connor K. Dementia after moderate-severe traumatic brain injury: coexistence of multiple proteinopathies. J Neuropathol Exp Neurol. (2018) 77:50–63. 10.1093/jnen/nlx101 29155947 PMC5939622

[3] Johnson VE Stewart JE Begbie FD Trojanowski JQ Smith DH Stewart W. Inflammation and white matter degeneration persist for years after a single traumatic brain injury. Brain. (2013) 136:28–42. 10.1093/brain/aws322 23365092 PMC3562078

[4] Mckee AC Stern RA Nowinski CJ Stein TD Alvarez VE Daneshvar DH. The spectrum of disease in chronic traumatic encephalopathy. Brain. (2013) 136:43–64. 10.1093/brain/aws307 23208308 PMC3624697

[5] Sandsmark DK Bashir A Wellington CL Diaz-Arrastia R. Cerebral microvascular injury: a potentially treatable endophenotype of traumatic brain injury-induced neurodegeneration. Neuron. (2019) 103:367–79. 10.1016/j.neuron.2019.06.002 31394062 PMC6688649

[6] Dams-O'connor K Seifert AC Crary JF Delman BN Del Bigio MR Kovacs GG. The neuropathology of intimate partner violence. Acta Neuropathol. (2023) 146:803–15. 10.1007/s00401-023-02646-1 37897548 PMC10627910

[7] Vande Vyvere T Pisica D Wilms G Claes L Van Dyck P Snoeckx A. Imaging Findings in acute traumatic brain injury: a National Institute of Neurological Disorders and Stroke common data element-based pictorial review and analysis of over 4000 admission brain computed tomography scans from the Collaborative European NeuroTrauma Effectiveness Research in Traumatic Brain Injury (CENTER-TBI) Study. J Neurotrauma. (2024) 41:2248–97. 10.1089/neu.2023.0553 38482818

[8] Merkley TL Bigler ED Wilde EA Mccauley SR Hunter JV Levin HS. Diffuse changes in cortical thickness in pediatric moderate-to-severe traumatic brain injury. J Neurotrauma. (2008) 25:1343–5. 10.1089/neu.2008.0615 19061377 PMC2747789

[9] Strangman GE O'neil-Pirozzi TM Supelana C Goldstein R Katz DI Glenn MB. Regional brain morphometry predicts memory rehabilitation outcome after traumatic brain injury. Front Hum Neurosci. (2010) 4:182. 10.3389/fnhum.2010.00182 21048895 PMC2967347

[10] Santhanam P Wilson SH Oakes TR Weaver LK. Accelerated age-related cortical thinning in mild traumatic brain injury. Brain Behav. (2019) 9:e01161. 10.1002/brb3.1161 30488646 PMC6346670

[11] Diamond BR Donald CLM Frau-Pascual A Snider SB Fischl B Dams-O'connor K. Optimizing the accuracy of cortical volumetric analysis in traumatic brain injury. MethodsX. (2020) 7:100994. 10.1016/j.mex.2020.100994 32760659 PMC7393399

[12] Dadar M Potvin O Camicioli R Duchesne S Alzheimer's Disease Neuroimaging Initiative. Beware of white matter hyperintensities causing systematic errors in FreeSurfer gray matter segmentations! *Hum Brain Mapp*. (2021) 42:2734–45. 10.1002/hbm.25398 33783933 PMC8127151

[13] Oi Y Hirose M Togo H Yoshinaga K Akasaka T Okada T. Identifying and reverting the adverse effects of white matter hyperintensities on cortical surface analyses. Neuroimage. (2023) 281:120377. 10.1016/j.neuroimage.2023.120377 37714391

[14] Fischl B. FreeSurfer. Neuroimage. (2012) 62:774–81. 10.1016/j.neuroimage.2012.01.021 22248573 PMC3685476

[15] Smith SM Jenkinson M Woolrich MW Beckmann CF Behrens TE Johansen-Berg H. Advances in functional and structural MR image analysis and implementation as FSL. Neuroimage. (2004) 23:S208–219. 10.1016/j.neuroimage.2004.07.051 15501092

[16] Friston KJ Holmes AP Worsley KJ Poline J-P Frith CD Frackowiak RSJ. Statistical parametric maps in functional imaging: a general linear approach. Hum Brain Mapp. (1994) 2:189–210. 10.1002/hbm.460020402

[17] Ding K De Marquez C La Plata JY Wang M Mumphrey C Moore C. Cerebral atrophy after traumatic white matter injury: correlation with acute neuroimaging and outcome. J Neurotrauma. (2008) 25:1433–40. 10.1089/neu.2008.0683 19072588 PMC2858299

[18] Warner MA Youn TS Davis T Chandra A De Marquez C La Plata C. Regionally selective atrophy after traumatic axonal injury. Arch Neurol. (2010) 67:1336–44. 10.1001/archneurol.2010.149 20625067 PMC3465162

[19] Kumar RG Selmanovic E Gilmore N Spielman L Li LM Hoffman JM. Distinct clinical phenotypes and their neuroanatomic correlates in chronic traumatic brain injury. Brain Commun. (2025) 7:fcaf216. 10.1093/braincomms/fcaf216 40574978 PMC12198765

[20] Iglesias JE Billot B Balbastre Y Tabari A Conklin J Gilberto Gonzalez R. Joint super-resolution and synthesis of 1 mm isotropic MP-RAGE volumes from clinical MRI exams with scans of different orientation, resolution and contrast. Neuroimage. (2021) 237:118206. 10.1016/j.neuroimage.2021.118206 34048902 PMC8354427

[21] Iglesias JE Billot B Balbastre Y Magdamo C Arnold SE Das S. SynthSR: A public AI tool to turn heterogeneous clinical brain scans into high-resolution T1-weighted images for 3D morphometry. Sci Adv. (2023) 9:eadd. 10.1126/sciadv.add3607 36724222 PMC9891693

[22] Edlow BL Keene CD Perl DP Iacono D Folkerth RD Stewart W. Multimodal characterization of the late effects of traumatic brain injury: a methodological overview of the late effects of traumatic brain injury project. J Neurotrauma. (2018) 35:1604–19. 10.1089/neu.2017.5457 29421973 PMC6016096

[23] U.S. Department of Defense. DoD Standard Surveillance Case Definition for TBI Adapted for AFHSB Use (2019). Available online at: https://www.health.mil/Reference-Center/Publications/2015/12/01/Traumatic-Brain-Injury (Accessed January 22, 2025).

[24] Van Der Kouwe AJW Benner T Salat DH Fischl B. Brain morphometry with multiecho MPRAGE. Neuroimage. (2008) 40:559–69. 10.1016/j.neuroimage.2007.12.025 18242102 PMC2408694

[25] Puonti O Iglesias JE Van Leemput K. Fast and sequence-adaptive whole-brain segmentation using parametric Bayesian modeling. Neuroimage. (2016) 143:235–49. 10.1016/j.neuroimage.2016.09.011 27612647 PMC8117726

[26] Cerri S Puonti O Meier DS Wuerfel J Muhlau M Siebner HR. A contrast-adaptive method for simultaneous whole-brain and lesion segmentation in multiple sclerosis. Neuroimage. (2021) 225:117471. 10.1016/j.neuroimage.2020.117471 33099007 PMC7856304

[27] Cerri S Greve DN Hoopes A Lundell H Siebner HR Muhlau M. An open-source tool for longitudinal whole-brain and white matter lesion segmentation. Neuroimage Clin. (2023) 38:103354. 10.1016/j.nicl.2023.103354 36907041 PMC10024238

[28] Soille P. Morphological Image Analysis. Berlin: Springer (2004).

[29] Jovicich J Czanner S Han X Salat D Van Der Kouwe A Quinn B. MRI-derived measurements of human subcortical, ventricular and intracranial brain volumes: reliability effects of scan sessions, acquisition sequences, data analyses, scanner upgrade, scanner vendors and field strengths. Neuroimage. (2009) 46:177–92. 10.1016/j.neuroimage.2009.02.010 19233293 PMC2866077

[30] De Vent NR Agelink Van Rentergem JA Huizenga HM Van Der Flier WM Sikkes WM Murre JMJ. An operational definition of ‘abnormal cognition' to optimize the prediction of progression to dementia: what are optimal cut-off points for univariate and multivariate normative comparisons? J Alzheimers Dis. (2020) 77:1693–703. 10.3233/JAD-200811 32925072 PMC7683061

[31] Reuter M Schmansky NJ Rosas HD Fischl B. Within-subject template estimation for unbiased longitudinal image analysis. Neuroimage. (2012) 61:1402–18. 10.1016/j.neuroimage.2012.02.084 22430496 PMC3389460

[32] Hoopes A Butoi VI Guttag JV Dalca AV. VoxelPrompt: A vision-language agent for grounded medical image analysis. arXiv. (2024) 2410:08397.

[33] Pollak C Kugler D Bauer T Ruber T Reuter M. (2025). FastSurfer-LIT: lesion inpainting tool for whole brain MRI segmentation with tumors, cavities and abnormalities. Imag Neurosci. 3:1–8. 10.1162/imag_a_00446 40109899 PMC11917724

[34] Adatia K Newcombe VFJ Menon DK. Contusion progression following traumatic brain injury: a review of clinical and radiological predictors, and influence on outcome. Neurocrit Care. (2021) 34:312–24. 10.1007/s12028-020-00994-4 32462411 PMC7253145

[35] Witcher KG Bray CE Chunchai T Zhao F O'neil SM Gordillo AJ. (2021). Traumatic brain injury causes chronic cortical inflammation and neuronal dysfunction mediated by microglia. J Neurosci 41:1597–616. 10.1523/JNEUROSCI.2469-20.2020 33452227 PMC7896020

[36] Scott G Zetterberg H Jolly A Cole JH De Simoni S Jenkins PO. Minocycline reduces chronic microglial activation after brain trauma but increases neurodegeneration. Brain. (2018) 141:459–71. 10.1093/brain/awx339 29272357 PMC5837493

[37] Edlow BL Tseng CJ Gilmore N Mckinney IR Tromly SL Deary KB. Neuroinflammation at the Gray-White Matter Interface in Active-Duty U.S. Special Operations Forces. Neurotrauma Rep. (2024) 5:1205–11. 10.1089/neur.2024.0116 39744610 PMC11685501

[38] Pinaya WHL Tudosiu PD Gray R Rees G Nachev P Ourselin S. Unsupervised brain imaging 3D anomaly detection and segmentation with transformers. Med Image Anal. (2022) 79:102475. 10.1016/j.media.2022.102475 35598520 PMC10108352

[39] Zoetmulder R Gavves E Caan M Marquering H. Domain- and task-specific transfer learning for medical segmentation tasks. Comput Methods Programs Biomed. (2022) 214:106539. 10.1016/j.cmpb.2021.106539 34875512

[40] De Rosa AP Benedetto M Tagliaferri S Bardozzo F D'ambrosio A Bisecco A. Consensus of algorithms for lesion segmentation in brain MRI studies of multiple sclerosis. Sci Rep. (2024) 14:21348. 10.1038/s41598-024-72649-9 39266642 PMC11393062

[41] Ma J He Y Li F Han L You C Wang B. Segment anything in medical images. Nat Commun. (2024) 15:654. 10.1038/s41467-024-44824-z 38253604 PMC10803759

[42] Monteiro M Newcombe VFJ Mathieu F Adatia K Kamnitsas K Ferrante E. Multiclass semantic segmentation and quantification of traumatic brain injury lesions on head CT using deep learning: an algorithm development and multicentre validation study. Lancet Digit Health. (2020) 2:e314–22. 10.1016/S2589-7500(20)30085-6 33328125

[43] Phaphuangwittayakul A Guo Y Ying F Dawod AY Angkurawaranon S Angkurawaranon C. An optimal deep learning framework for multi-type hemorrhagic lesions detection and quantification in head CT images for traumatic brain injury. Appl Intell. (2022) 52:7320–38. 10.1007/s10489-021-02782-9 34764620 PMC8475375

[44] Macintosh BJ Liu Q Schellhorn T Beyer MK Groote IR Morberg PC. Radiological features of brain hemorrhage through automated segmentation from computed tomography in stroke and traumatic brain injury. Front Neurol. (2023) 14:1244672. 10.3389/fneur.2023.1244672 37840934 PMC10568013

[45] Kamnitsas K Ledig C Newcombe VFJ Simpson JP Kane AD Menon DK. Efficient multi-scale 3D CNN with fully connected CRF for accurate brain lesion segmentation. Med Image Anal. (2017) 36:61–78. 10.1016/j.media.2016.10.004 27865153

[46] Edlow BL Mareyam A Horn A Polimeni JR Witzel T Tisdall MD. 7 Tesla MRI of the ex vivo human brain at 100 micron resolution. Sci Data. (2019) 6:244. 10.1038/s41597-019-0254-8 31666530 PMC6821740

